# Towards Digital Twin Implementation for Assessing Production Line Performance and Balancing †

**DOI:** 10.3390/s20010097

**Published:** 2019-12-23

**Authors:** Marcello Fera, Alessandro Greco, Mario Caterino, Salvatore Gerbino, Francesco Caputo, Roberto Macchiaroli, Egidio D’Amato

**Affiliations:** 1 Department of Engineering, University of Campania Luigi Vanvitelli, via Roma 29, 81031 Aversa, Italy; marcello.fera@unicampania.it (M.F.); mario.caterino@unicampania.it (M.C.); salvatore.gerbino@unicampania.it (S.G.); francesco.caputo@unicampania.it (F.C.); roberto.macchiaroli@unicampania.it (R.M.); 2 Department of Science and Technology, University of Napoli Parthenope, Centro Direzionale Isola C4, 80143 Naples, Italy; egidio.damato@uniparthenope.it

**Keywords:** wearable devices, Internet of Things—IoT, methodological framework, simulation, production line performance

## Abstract

The optimization of production processes has always been one of the cornerstones for manufacturing companies, aimed to increase their productivity, minimizing the related costs. In the Industry 4.0 era, some innovative technologies, perceived as far away until a few years ago, have become reachable by everyone. The massive introduction of these technologies directly in the factories allows interconnecting the resources (machines and humans) and the entire production chain to be kept under control, thanks to the collection and the analyses of real production data, supporting the decision making process. This article aims to propose a methodological framework that, thanks to the use of Industrial Internet of Things—IoT devices, in particular the wearable sensors, and simulation tools, supports the analyses of production line performance parameters, by considering both experimental and numerical data, allowing a continuous monitoring of the line balancing and performance at varying of the production demand. A case study, regarding a manual task of a real manufacturing production line, is presented to demonstrate the applicability and the effectiveness of the proposed procedure.

## 1. Introduction

As introduced by the authors in [1], in recent years, industries are going through a period of massive changes, named Industry 4.0—I4.0, aimed to achieve a higher level of operational efficiency and productivity [2] and to provide resources able to enhance the products and production lines quality with a focus on costs reduction too. Within this context, factories are becoming smart, more flexible and collaborative, in order to satisfy the current demands of an increasingly competitive market. This objective can be reached by implementing some enabling technologies of I4.0 (Cyber Physical Systems—CPS, Internet of Things—IoT, Cloud Computing, Augmented Reality—AR, Big Data analytics, Simulation, etc.) that will increase the production speed, reducing the costs connected to errors or stops of production. Furthermore, these technologies allow avoiding complex mathematical problems, such as Non-determistic Polynomial-time hard (NP-hard), typical for production line scheduling and balancing models [3].

This paper is focused on the use of IoT and simulation tools that, by integrating sensors and simulators for investigating human working performance [4], allow in-line adjustments and process control, enhancing a near-real time management of the production lines based on different kinds of production technologies [5]. IoT, despite the different visions of the scientific communities [6], is considered as a pervasive presence around us of a variety of things or objects (sensors, actuators, mobile phones, etc.), which, through unique addressing schemes, are able to interact with each other and cooperate with their neighbors to reach common goals. Thanks to these technologies, it is possible to connect different devices and get them to communicate. Among these, wearable devices are very important since they are provided with sensors capable to measure movements, lights, sounds, temperatures, etc. and to communicate these data in real, or near-real, time to other devices or users. In this way, it is possible to continuously monitor the desired parameters [7] and their performance regarding the implemented optimization strategy, such as scheduling or balancing [8,9].

Wearable devices can be used for evaluating and monitoring the parameters that contribute to the work-related biomechanical load. Among these parameters, working postures are the main cause of Musculo-Skeletal Disorders—MSD [10]. Thanks to wearable sensors, it is possible to collect data about the postures assumed by workers during a working activity. The processing of these data allows understanding how to improve ergonomics, working times, etc. [11,12]. Moreover, these devices can be integrated with other wearables, such as Surface Electromyography—EMGs electrodes, for studying how different parameters can be related to each other [13,14,15]. Wearable devices offer the possibility to analyze data related both to real production phases and pre-production phases, as well as to the design phase, when the physical production line does not exist yet, providing in this way preventive information about the production line performance factors.

In manufacturing working scenarios, in addition to IoT applications, an important aspect is the possibility to fully reproduce on computer the actual environment being tested. This allows simulating the operations of a real-world process [16] and making possible, already at the design phase, choosing the correct solutions affecting the operating conditions of a production system: it is the so-called Digital Twin—DT. Computer simulations are used as useful tools for both validating the design of production processes and evaluating performance parameters of production lines in near real time. The DT becomes as a digital controller of the real world manufacturing system.

### 1.1. Previous Researches

About the performance evaluation of a production line via simulation, a lot of work has been done in the scientific community. Fisher and Ittner [17] evaluated the impact of product variety on automobile assembly plant performance. Savsar [18] used simulation approach to determine the minimum number of batches in an electronic line assembly. Gujarathi et al. [19] were able to carry out a production capacity analysis in a shock absorber assembly line thanks to simulation and then, by analyzing the results, they proposed a modification of the line. Di Gironimo et al. [20] developed a discrete event simulation to optimize the planning of a high-speed train production system. Sandanayake et al. [21] applied computer-based simulation tools and linear mathematical models to identify the impact of selected Just In Time—JIT parameters on performance in an automotive component manufacturing environment. Nagarur and Azeem [22] studied how the part commonality and machine flexibility affected typical parameters of a manufacturing system (such as makespan, machine utilization, and factor productivity) by means of simulation. In [23,24,25], the authors present three DT-based methodologies for supporting the rapid design and the control of smart manufacturing systems, focusing also on the synchronization between the physical and the digital worlds.

In addition, it is worth to note that manual operations are still dominant in complex manufacturing processes [26] so the working tasks results are affected by high variability. For these reasons, it is interesting to investigate the possibility to implement real data into a simulation, via Digital Twin, in order to speed up the working time analyses or ergonomics assessments, currently carried out manually and in an observational way, hence by means of time consuming processes.

In the literature, it is possible to find several researches about digital human modeling and simulation approaches, especially for ergonomic evaluations. In [27,28], Caputo et al. present a numerical/experimental procedure for evaluating ergonomic performance useful for both designing new workplaces and monitoring the working activity during the real production. About the study of working postures, De Magistris et al. [29] analyzed joint angles of Digital Human Models—DHM for assessing ergonomic indexes, while Alexoupolos et al. [30] developed a so-called ErgoToolkit for postures recognition and ergonomic evaluation. Björkenstam et al. [31] used Discrete Mechanics and Optimal Control—DMOC method to optimize trajectories in manual lifting operations. Case et al. [32] studied the workers’ ageing by implementing human capability data within a simulation environment. In industrial environment, human simulations can facilitate the assembly processes, improve the product and production process development in a cost and time effective approach and improve ergonomics since the early design phase.

However, these numerical simulations may provide inaccurate results since human motion is evaluated by inverse kinematic, as used in robotics, letting the virtual manikin move more similar to a robot than a human. The implementation of real humans’ behavior in DHM can allow accurate simulations and assessment of working times and human performances.

Very few significant works have been documented in the technical literature about simulation based on experimental data, collected in a real working environment by wearable motion tracking systems and most of them are relative to the control of human body for clinical purposes and not to evaluate workers’ performances.

Mukhopadhyay in 2015 [33] reviewed the systems on activity monitoring of humans based on wearable sensors and explained how the data collected may be post-processed by means of simulation. Knight et al. [34] demonstrated how accelerometers mounted on a wearable system may be used to collect data in order to assess human performances in a work environment. Catarci et al. [35] provided a detailed literature review about DTs and their modeling techniques. Moreover, they proposed a complex DT architecture for digital factories. Malik and Bilberg [36] presented the DT of a human-robot collaboration work-cell based on event-based simulation that continuously updates the changes of real counterpart, without transferring real kinematic data to the digital human. A detailed description of digitalization processes of factories, according to the Industry 4.0 paradigm, is proposed by Belli et al. [37], together with an application developed for monitoring the production and the quality control. Short and Twiddle [38] developed the digitalization of a water industry application, while Li et al. [39] developed an AR application for the control and adjustment of robots during a human-robot interaction based working task. They implemented the DT of human hands by means of LeapMotion sensor and a Kinect V2 camera.

An interesting study about the implementation of DT based on sensor data fusion and motion recognition of human activity and simulation has been proposed by Nikolakis et al. [40]. They used experimental data for generating realistic simulations mainly for ergonomic analysis.

### 1.2. Purpose and Article Outline

This paper is aimed to propose a novel methodological framework, partially presented in [1], based on the implementation of human motion data collected by a wearable motion tracking system in a numerical simulation, tending to a Digital Twin. The proposed procedure allows carrying out near real time analyses capable to provide feedbacks about the production line efficiency, giving the possibility to team leaders to modify and optimize the balancing or the assignment of the working tasks during the real work shift.

A preliminary case study, concerning a real working task, has been carried out at the Laboratory of Machine Design of the University of Campania Luigi Vanvitelli. Experimental data have been acquired by using a wearable inertial motion tracking system, prototyped at the same laboratory [10,11], while the DT has been implemented in Tecnomatix Process Simulate by Siemens^®^ virtual environment.

The reminder of this paper is organized as follows. Section 2 describes the methodological framework, aimed to support the assessment of production line performance, based on the Digital Twin of the working environment. Section 3 describes the real case study that investigates a real workstation of a manufacturing production line, with a detailed discussion about the data collection and implementation in the numerical simulation. Section 4 presents the results analysis and the consequent discussion, while Section 5 concludes the paper.

## 2. Methodological Framework

**Figure 1:**
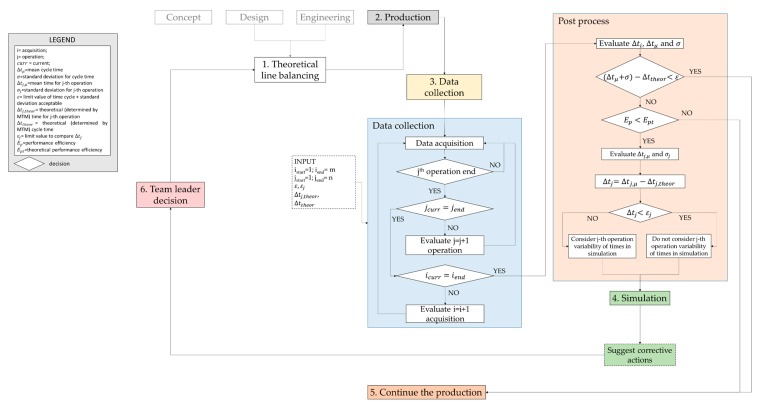
General methodological framework for evaluating the line performance during the production.

The methodological framework developed to support the improvement of the performance and the balancing of a production lines, which involve manual work, is presented in Figure 1. The proposed model aims at:

- transferring experimental data collected during the real production to a simulation environment;
- evaluating specific parameters such as cycle time, workers’ saturation, etc., and measuring the Performance Efficiency—E of the production line by implementing the experimental data in the simulation environment;
- comparing the results with the nominal values, defined during the design phase, when experimental data were not available;
- transferring the results of the simulation to production line manager who will have the possibility to modify and re-balance the line.

In detail, the framework has been designed for manufacturing factories that want to evaluate the significant parameters of their production lines by using the simulation as testing and decision support tool for verifying the effectiveness of the initial, or on-going, line balancing and job assignment. So, in the present paper, the design phase of the process is not considered. Design parameters, such as cycle time, workers’ saturation, line efficiency, product quality, etc., will be assumed as an input of the procedure. The simulation may also provide useful information for revising the design phase if the corrective measures are not applicable.

The first step of the framework (Figure 1) considers the nominal balancing of the line (box 1 in Figure 1), based on the previous experience of similar lines and on time analysis methods, already defined during design and engineering phases. During the production process (box 2 in Figure 1), the procedure suggests to investigate if the nominal balancing is verified. Hence, data collection (box 3 in Figure 1), related to working times, is required to evaluate line performance factors. If necessary, collected data are transferred and implemented in an event-based simulation (box 4 in Figure 1) that allows evaluating the performance parameters during a whole working shift. Numerical results analysis could suggest corrective measures for improving the line balancing and scheduling.

The following sections describe in detail the data collection and simulation steps, which represent the core of the general methodological framework of Figure 1.

### 2.1. Data Collection

Data collection (box 3 in Figure 1) is one of the most important points of the proposed approach. In the present study, data collection is relative to the capture of human body movements.

There are several human motion capture systems, divided into two main groups, which can be used:

- Optical systems: they consist of cameras installed within the test environment that capture the position of on-body sensors (in most cases they are simply markers) and allow to reproduce movements. They are mainly used in the field of biomechanics to record human motion because of their high accuracy and their capacity of real time preview of the motion, even if they are very expensive and bulky to be used in production lines.
- Non-Optical systems: all the other systems that do not use cameras may be classified in this category. Depending on the type of used sensors, there are several devices useful to acquire human body postures, such as:
  (i.) Electromechanical system, mainly consisting in wearable suites with wires and joints. This device is cheaper than the optical one, but it is less accurate and it obstructs the user’s movements.
  (ii.) Magnetic system, which uses magnetic sensors to evaluate the position and the directions of human body segments with respect to a magnetic field generator. The problem of this device is that the magnetic field generator cannot work in many real production environments because of magnetic interferences. So, magnetic systems can be used only in a laboratory environment, keeping the sensors near to the magnetic field generator.
  (iii.) Inertial Movements Unit (IMU) sensors, which integrate magnetometer, accelerometer, and gyroscope to capture the orientation of each segment of human body. Inertial sensors are worn by the user and, usually, they do not obstruct the working activities; moreover, as opposed to magnetic sensors, IMU sensors can be used in a real working environment, even if they require an accurate initial calibration. Finally, inertial motion tracking systems are cheaper than the optical systems, but the accuracy is limited and the measurements may also be affected by electromagnetic noise due to the presence of metals in a real factory environment.

The inertial motion tracking systems are the most suited ones for acquiring data in factory environment. Whatever motion tracking system is chosen, a calibration phase is required in the acquisition environment.

#### 2.1.1. Number of Acquired Cycles

This parameter is needed because, as it will be explained in next paragraphs, data are processed in order to obtain the mean and standard deviation values of working times. The number of acquisitions strictly depends on the cycle time of the working task: in particular, the smaller the cycle time, the greater the number of acquisitions will be because the variability of acquisition increases [41].

#### 2.1.2. Data Acquisition Session

Once the sensors have been calibrated, the number of acquisitions has been defined, and the worker has worn the sensors, the data acquisition session starts.

In order to evaluate whether the chosen system interferes with the working activities, at the end of the acquisition phase, a questionnaire, concerning the usability of the system, could be submitted to the workers. In case the interviews highlight the system is bulky and interferes with the working task, it is possible to introduce a corrective coefficient depending on the level of obstruction and on the kind of working task.

When data acquisition ends, data collected must be processed to define the mean and the standard deviation values of real cycle time.

#### 2.1.3. Post Process

As said before, data coming from acquisition sessions are relative to human motion. According to the procedure depicted in Figure 1, it would be desirable to use a system that analyzes data autonomously, able to recognize the working cycles (*i*) and the operations (*j*) in order to evaluate working times. In that case, the phases of data collection (blue box in Figure 1) and post process (orange box in Figure 1) can be carried out automatically by the system (the user should only set the number of acquisition and define the number of operations in which the cycle is divided). When data acquisition starts, the system should be able to monitor the progress of operations and working cycles and to stop the data collection when the last operation of the last cycle is performed.

At the end of data acquisition, the post process phase starts and data are analyzed (orange box of Figure 1).

Since this framework is focused on performance line parameters, the working times, per each acquired cycle, can be evaluated and compared with those ones estimated by Method and Time Measurements—MTM analysis, carried out during the production process engineering phase. In detail, it is possible to divide the entire working activity in *j* sub-operations, defined according to the variability of tasks, to the different methods used by workers to carry out the operation, or to the length of the entire cycle. So, defining:

- *t*_0*i*_ = timeframe corresponding to the start of the *i*th production cycle;
- *t*_1*i*_ = timeframe corresponding to the end of the *i*th production cycle.

The difference between these two timeframes provides the working cycle time for the *i*th cycle (*WCt_i_*):

(1) $$WCt_{i}=t_{1i}-t_{0i}=\Delta t_{i}$$

naming *OP_j_* the operations of the working cycle (*WC*), this will be defined as the sum of the single operations:

(2) $$WC=\overset{n}{\underset{j=1}{\sum }}OP_{j}$$

where *n* is the number of operations composing the working cycle.

The time for carrying out each operation needs to be monitored. So, by defining:

- ∆*t_ij_* = time needed for the *j*th operation in the *i*th acquisition;
- *t*_0*ij*_ = timeframe corresponding to the start of the *j*th operation in the *i*th acquisition;
- *t*_1*ij*_ = timeframe corresponding to the end of the *j*th operation in the *i*th acquisition;
- it will be:

(3) $$\Delta t_{ij}=t_{1ij}-t_{0ij}$$

and:

(4) $$WCt_{i}=\overset{n}{\underset{j=1}{\sum }}\Delta t_{ij}$$

To evaluate whether a simulation is needed to assess the line performance, the procedure in Figure 1 proposes two levels of screening: the first level, based on the evaluation of working cycle times ∆*t_i_*; the second level, based on the evaluation of the working times for each operation ∆*t_ij_*.

As shown in Figure 1, the data collection step begins when the production is already started, i.e., when the working tasks are already assigned. This implies that, during the engineering phase, a time and method analyst has evaluated the theoretical time value ∆*t_j_*_,*theor*_ for carrying out the *j*th operation. ∆*t_j_*_,*theor*_ can be evaluated by using several methods including the Method Time Measurement—MTM.

By comparing the experimental time values, evaluated by analyzing collected experimental data, and the theoretical time values estimated by the analyst, a first screen of the line performance can be obtained in order to establish whether a simulation is needed or not. In particular, two levels of assessment are proposed as follows.

*Level 1*: the comparison concerns the mean experimental cycle time value ∆*t_μ_* (inclusive of standard deviation *σ*) of the entire working cycle and the theoretical cycle time value ∆*t_j_*_,*theor*_. The mean cycle time ∆*t_μ_* and the standard deviation *σ* can be derived by the following Equations (5) and (6):

(5) $$\Delta t_{\mu }=\frac{\sum_{i=1}^{m}\Delta t_{i}}{m}$$

(6) $$\sigma =\;\sqrt{\frac{\sum_{i=1}^{m}{\left(\Delta t_{i}-\Delta t_{\mu }\right)}^{2}\;}{m}}$$

where *m* is the total number of acquired cycles.

According to inequality (7), if the difference between ∆*t_μ_* and ∆*t_j_*_,*theor*_ is less than a threshold value *ε*, defined by the production engineers, it is possible to argue that no problem affects the production system and production can continue. In this case, no simulation is needed and the line does not need corrective actions. The threshold value *ε* is set by engineers, based on the type of activities.

(7) $$\left|\left(\Delta t_{\mu }+\sigma \right)-\Delta t_{theor}\right|<\varepsilon$$

If the inequality (7) is not verified, the performance efficiency *E_p_* may be considered and compared to its estimated value *E_p_*_,*t*_. In case of a completely manual workstation, *E_p_* and *E_p_*_,*t*_ are considered as the number of produced components during the working shift and its estimated value respectively. So, if the following inequality (8) is verified, the production can continue. Otherwise, the second level screening is necessary.

(8) $$E_{p}\geq E_{p,t}$$

*Level 2*: this screening is necessary to point out, by means of simulation, which operations may be affected by critical issues. The first step is to evaluate the mean experimental time value $\Delta t_{j,\mu }$ and the standard deviation $\sigma_{j}$ for each *j*th operation, by using the following Equations (9) and (10):

(9) $$\Delta t_{j,\mu }=\frac{\sum_{i=1}^{m}\Delta t_{ij}}{m}$$

(10) $$\sigma_{j}=\sqrt{\frac{\sum_{i=1}^{m}{\left(\Delta t_{ij}-\Delta t_{j,\mu }\right)}^{2}\;}{m}}$$

When these values are known, it is possible to compare the difference between ∆*t_j_*_,*μ*_ and its theoretical value ∆*t_j_*_,*theor*_ to a threshold value *ε_j_*, related to the *j*th operation, by verifying the following inequality (11):

(11) $$\Delta t_{j}=\left|\Delta t_{j,\mu }-\Delta t_{theor,j}\right|<\varepsilon_{j}$$

If verified, it is possible to suppose that the *j*th operation does not contribute to line performance issues and so the time variability of this operation will not be considered in the simulation stage. Otherwise, if the inequality (11) is not verified, the *j*th operation is one of the possible cause of the line problem and so it must be deeply investigated in the simulation stage. In particular, the time variability will be implemented in an event based simulation that, combining all the possible solutions given by its mean ∆*t_j_*_,*μ*_ and its standard deviation *σ_j_* time values, will provide information on the criticalities of the production line.

### 2.2. Simulation

**Figure 2:**
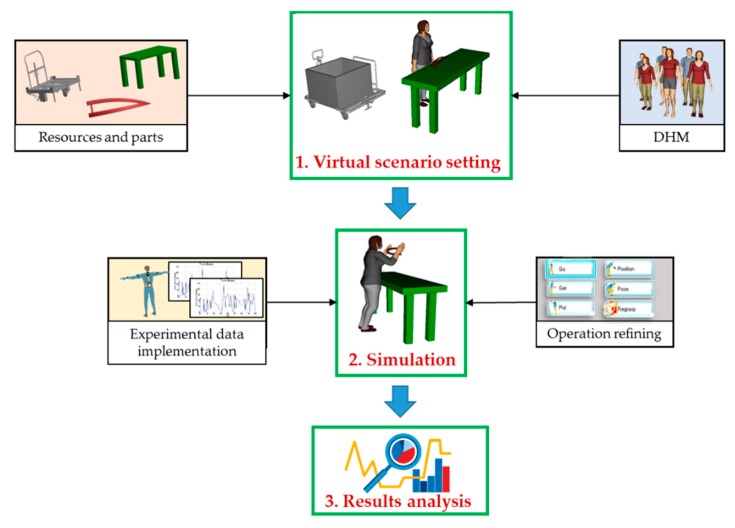
Procedure for realizing the simulation of the investigated working activity.

Figure 2 shows the procedure for realizing a simulation of manual working tasks, composed by three main steps: virtual scenario setting, simulation, and results analysis.

- **Virtual scenario setting**: the first step regards the virtual representation of the investigated workstation. Having 3D models of resources and parts, it is possible to set up the virtual scenario according to the design specifications. Once the objects are positioned, the male/female DHM is created and customized according to the desired anthropometric characteristics.
- **Simulation**: the software for human simulation, such as Tecnomatix by Siemens^®^ [42] or Delmia by Dassault Sistèmes^®^ [43], allows creating operations (walking, reaching, gripping, positioning, assuming a posture, applying force…) in a very intuitive way. These environments allow integrating motion capture systems (such as Kinect^®^, Vicon^®^, or XSens^®^), with already implemented communication interfaces, to connect the DHM with the user, replicating the motion. In addition, they give the possibility to build an own interface in order to connect any motion capture system. Since, by using these devices, it is possible to replicate the only motion, it may be necessary to refine other operations, such as grasping, picking/placing, or handling of objects. Simulating an entire work shift, by means of event-based simulation, allows evaluating the performance parameters of the line (working times, number of produced items…), ergonomics and so on.
- **Results analysis**: numerical data, related to the line performance parameters, need to be analyzed and evaluated in order to deal with the decision-making process, suggesting corrective measures to the team leader.

## 3. Case Study

In order to show the effectiveness of the methodological framework depicted in Figure 1, a case study regarding a real working activity is described below. It is related to a workplace of a production line of an automotive components manufacturer, in particular headlamp plastics. The investigation concerns a workplace in which a worker manually performs a quality control of a car’s component.

**Figure 3:**
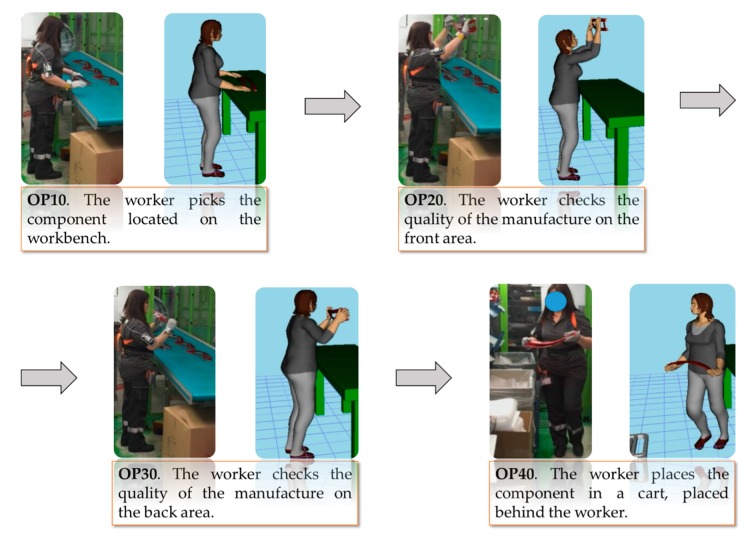
Tasks of the working activity. On the left is the real scenario and on the right is the Digital Twin.

Figure 3 shows the four tasks characterizing the working activity. On the left side of each block the real working scenario is depicted; on the right the Digital Twin.

According to the framework in Figure 1, the first step of the procedure considers the nominal balancing of the line, already estimated during the design and engineering phases of the production system.

### 3.1. Data Collection

Knowing the line balancing parameters and once the working activities during the production have been analyzed (box 2 of Figure 1), for step 3—data collection, experimental data have been collected by using a wearable inertial motion tracking system developed at the Department of Engineering of the University of Campania Luigi Vanvitelli [10,11]. It has been realized in collaboration with the company Linup^®^ Srl in order to provide a low-cost system able to evaluate the attitude of the desired body segments and the posture angles of workers during the working activities in manufacturing production systems. It is composed, in upper body configuration, by two independent modules, properly synchronized during the data acquisition. Four IMUs, located at the pelvis, the trunk, the arm, and the forearm, respectively, compose each module that is connected to a Raspberry Pi, where raw data are recorded. The upper body configuration, which requires two modules, means that the sensors on the trunk and pelvis are redundant. Sensors data are transferred to a personal computer via Wi-Fi and then processed by a motion tracking algorithm, which uses a Kalman Filter for estimating attitude data at 100 Hz. CSV files are autonomously compiled by the algorithm, providing Euler angles, quaternions, and posture angles over the time for each considered segment.

**Figure 4:**
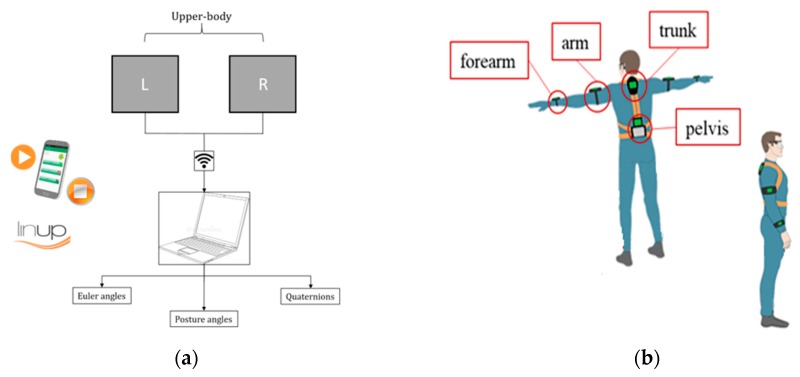
Wearable motion tracking system in upper-body configuration: architecture (**a**) and wearable suite (**b**).

In Figure 4, it is possible to observe the scheme of the used wearable motion tracking system.

During the step 3, according to Giacomazzi [41], experimental data have been acquired for 73 consecutive working cycles (*i* = 73). Furthermore, the parameters *ε* and *ε_j_* have been defined, while the ∆*t_theor_* is already known because it is defined during step 1 (box 1 of Figure 1) of the procedure.

**Figure 5:**
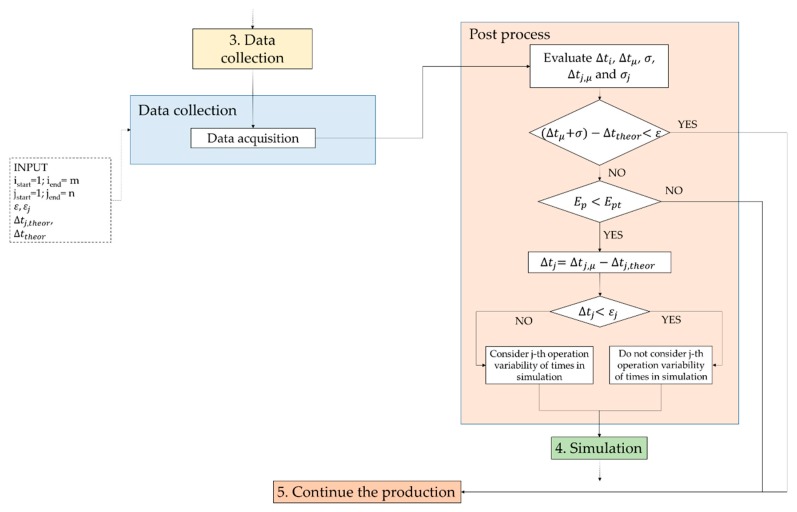
Modified procedure for data collection and post process.

It is worth noting that the post-process phase is not still carried out automatically and it has been performed manually. In detail, since the automatic recognition of manual working tasks is an open issue, during the data collection, triggers have been introduced to define both the starting and ending timeframe of each operation. So, the post process phase has been modified as shown in Figure 5.

When data acquisition ends, the procedure provides for the evaluation of mean experimental cycle time (∆*t_μ_*), and its standard deviation (*σ*), in order to compare them with the estimated cycle time (∆*t_theor_*), according to inequality (7).

**Figure 6:**
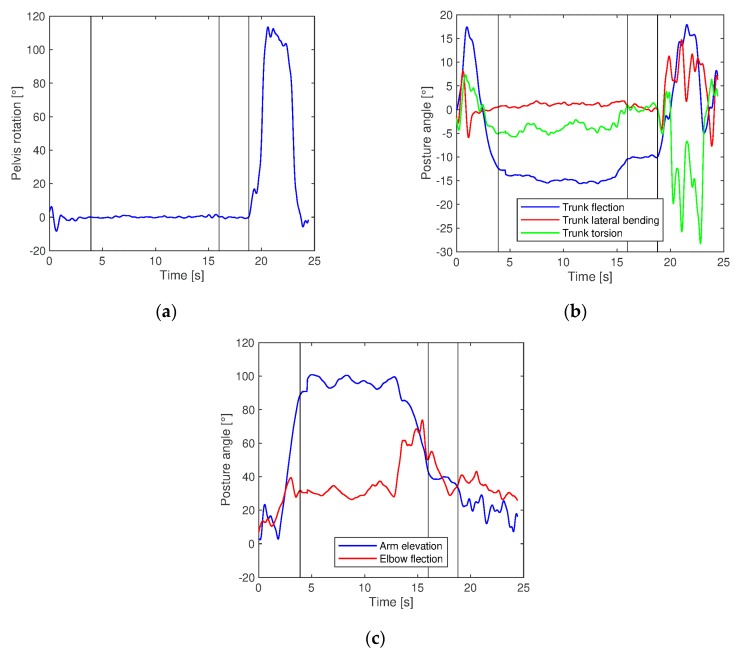
Posture angle trend for pelvis (**a**), trunk (**b**), and right limb (**c**) over one working cycle and triggers operations splitting.

Figure 6 shows the main posture angles for pelvis, trunk, and right limb and the triggers inserted for determining the start of each operation.

As triggers per each operation are introduced in the acquisition, the values ∆*t_μ_* and *σ* have not been evaluated by using Equations (5) and (6), as suggested by the framework of Figure 1. As it is possible to consider the operations s-independent, ∆*t_μ_,* and *σ* have been evaluated by the following equations:

(12) $$\Delta t_{\mu }=\overset{n}{\underset{j=1}{\sum }}\Delta t_{j,\mu }$$

(13) $$\sigma =\sqrt{\overset{n}{\underset{j=1}{\sum }}\sigma_{j}^{2}}$$

Table 1 summarizes the experimental and theoretical time values of *j* operations.

The parameter *ε*, in this case study, has been considered equal to the 15% of ∆*t_theor_*, i.e., 3 s.

It is possible to assess the first level screening by using inequality (7), considering ∆*t_μ_* = 21.60 s, *σ* = 3.08 s, and ∆*t_theor_* = 20 s.

The inequality (7) is not verified; so, it is reasonable to suppose the production line is affected by some issues. In order to verify this assumption, the framework of Figure 1 suggests the evaluation of the efficiency of workers (*E_p_*), related to the number of controlled items, and its comparison with its theoretical value (*E_p_*_,*t*_), according to inequality (8). During the data acquisition time range, the *E_p_*_,*t*_ is equal to 86, while the actual controlled items, *E_p_* are 73. *E_p_ < E_p_*_,*t*_, the inequality (8) is not verified and the second level screening is needed.

An event based simulation, which considers time variability of the operations, needs to be performed. In order to limit the computational times, the time variability is introduced only for the most critical operations, i.e., for those ones that do not respect the inequality (11).

Table 2 resumes the results obtained by applying inequality (11). The value of *ε_j_* has been considered equal to the 10% of the estimated time ∆*t_j_*_,*theor*_. It is worth noting that the values of *ε* (referred to the entire cycle) and *ε_j_* (referred to the single operations) are different because inequality (7) considers the standard deviation.

According to the framework, only the times variability related to OP20 and OP30 have to be implemented within the simulation. This agrees with the assumption that time variability is expected only for quality control operations (OP20 and OP30) and not for pick and place operations (OP10 and OP40), characterized by high repeatability.

### 3.2. Simulation

Concerning the simulation, the environment chosen for carrying out step 4 of the proposed framework—Digital Twin and Simulation, is Tecnomatix Process Simulate v15.0.1 by Siemens^®^. The real working scenario has been reproduced by using the 3D models of resources and parts of the real production line. The experimental human motion data have been considered as kinematic input, in terms of attitude angles of segments with respect to reference frames located at the joints, for a DHM, whose anthropometry has been customized according to the real worker, as shown in Figure 3.

The virtual environment has been augmented with a custom plugin to load sensors data, stored during the test campaign. Indeed, to make the virtual environment compliant with sensors data, a custom plugin (based on so-called Viewer objects in Tecnomatix Process Simulate) has been developed in Visual C#. It gives the ability to specify in the software user interface the name of a CSV text file where the sensors data are stored during the line tests.

The viewer is able to create compound operations based on poses store in a CSV text file, by using the “CreateHumanCompoundOperation” function of the root compound object.

In this simulation, only data about joint angles of trunk, shoulders, and elbows have been used.

The pseudocode (Algorithm 1) about the implemented procedure is shown below, recalling objects and functions used in C# NET framework.

**Algorithm 1** Pseudocode to link virtual environment with sensors data  Load data from CSV file  cocd=new TxCompoundOperationCreationData(“Root Folder”,1,”root”)  rootcompound=TxApplication.ActiveDocument.OperationRoot.CreateCompoundOperation(cocd)  For each line in CSV c_opcd=new TxHumanCompoundOperationCreationData c_op=rootcompound.CreateHumanCompoundOperation(c_opcd) c_ppcd=new TxHumanPostureOperationCreationData for each sensor in current CSV line  set posture by using human.SetJointAngles end c_pp=c_op.CreateHumanPostureOperation link c_pp with previous posture operation (using IncomingLinks and OutgoingLinks properties of c_pp)  end

Other details of the simulation, such as grasp and place operations, have been refined manually.

About the motion, the experimental data related to the working cycle, among those acquired, characterized by the cycle time (∆*t_i_*) whose value is closest to the theoretical one (∆*t_theor_*), have been implemented in the digital twin.

A working shift of 7.5 h (27,000 s), excluding breaks, has been simulated, implementing the time variability on the operations OP20 and OP30, as described in the previous section.

## 4. Results Analysis and Discussions

For the investigated case study, numerical data analysis focused on the evaluation of the number of working cycles (k) whose cycle time ∆*t_k_* exceeds the ∆*t_theor_*, equal to 20 s, in an entire working shift.

It has been considered that the production line is affected by critical issues if the cycle time ∆*t_k_* exceeds the ∆*t_theor_* for more than the 50% of the shift. The results are described in the following Table 3.

83.4% of the simulated cycles are characterized by ∆*t_k_ >* ∆*t_theor_*, proving the hypotheses made during the data post process phase (Section 3.1). In fact, defining ∆*t_μ_*_,*s*_ as the mean working cycle time in simulation, it is:

$$\Delta t_{\mu ,s}=\frac{Total\;simulation\;time\;\left[s\right]}{Number\;of\;completed\;cycles\;\left[k\right]}=\frac{27000}{1205}=22.4\;\left[\frac{s}{k}\right]$$

Table 4 shows the mean working cycle times.

So, it is verified that:

$$\Delta t_{\mu ,s}>\Delta t_{\mu }>\Delta t_{theor}$$

Since the numerical mean cycle time (∆*t_μ_*_,s_) value is higher than both the theoretical (∆*t_theor_*) and the experimental (∆*t_μ_*) values, it represents a worsening situation compared to that one estimated at the design stage of the line.

Consequently, the framework, in Figure 1, suggests to share the results with the team leader and to propose corrective measures, such as to employ another worker on the same work station, aimed to improve the line balancing and, hence, the whole production process.

It is worth pointing out that the computational cost for simulating a whole working shift has been about 4 h, by using a Z840 Hewlett-Packard computer with Intel Xeon CPU E5-2620 v3 at 2.40 GHz. This is mainly due to data sampling that needs to be optimized as a function of the desired output. If the purpose is to evaluate parameters related to working times, as in the present case, the sampling time should be increased, speeding up the simulation. On the other hand, if the purpose is the ergonomic assessment, the detail level needs to be higher, so the sampling time should be decreased, slowing down the simulation. In addition, other improvements will focus on increasing the speed of experimental data transfer.

Concerning the proposed procedure, as demonstrated, the purpose is not limited to the replication of human motion but mainly in the measurement of production systems performance. Since the acquired data need to be processed before performing the simulation, a timespan is required between the experimental data acquisition and the start of the simulation, when required. This time could be reduced if the motion tracking system is able to process row data simultaneously with their acquisition, merging the data collection (blue box of Figure 1) and post process (orange box in Figure 1) steps and automating part of the whole procedure.

## 5. Conclusions

This paper presents a novel methodological framework for the evaluation of production line performance based on real data coming from wearable sensors and Digital Twin. The procedure proposes the use of motion tracking systems for data acquisition once the production has already started, allowing a comparison between the working times, evaluated during the real production, and those ones estimated during the line design phase. A simulation, based on the Digital Twin of the real workstation, may be performed in order to figure out which operations cause production problems. In order to show the effectiveness of the proposed framework, a case study has been developed, in which a quality control operation is carried out. Data about human postures and working times have been acquired by means of a wearable motion tracking system based on IMUs. Issues related to worker performance deterioration, e.g., due to fatigue, have been neglected for this research.

It is worth to highlight that the procedure used in the case study was different from that one proposed in the general framework of Figure 1. In fact, the whole working cycle needs to be split in sub-operations in order to evaluate mean and standard deviation values of the working times. According to the framework, these steps could be carried out automatically by the system once the number of acquisitions and operations have been defined. In the presented case study, data acquisition and post-process stages have been conducted manually thanks to the introduction of triggers in data set, since the technologies for the automatic recognition of operations were not available for the performed experimental campaign.

In the case study, the Digital Twin has been realized by implementing experimental human motion data to a Digital Human Model by means of an ad-hoc interface between the software environment and the motion tracking data. Then, an event-based simulation has been carried out to evaluate if corrective actions were required.

The numerical results have shown that critical issues due to the high variability of two operations, which cause production delays, affect the line. Based on these feedbacks, the team leaders could introduce corrective measures to improve the performance and tend to the nominal balancing, as estimated during the production line design.

It is fundamental to underline that all the ethical requirements about the use of tracking devices must be respected, according to the national laws. Motion tracking systems must be used only for a limited timespan with the aim to investigate and improve the working conditions and the production performance and not to monitor the worker. In any case, the worker must be properly informed about the activity and they have to agree to data management.

This paper represents a first step for the development of the proposed methodological framework. Future works will be focused on the definition of methods to make the system able to automatically recognize the operations and to approach the goal of monitoring line performance in real time.

## Figures and Tables

**Table 1:** Time values for working cycle operations.

	∆*t_j_*_,*μ*_ [s]	*σ_j_* [s]	∆*t_j_*_,*theor*_ [s]
OP 10 (*j* = 1)	2.30	0.10	2.30
OP 20 (*j* = 2)	10.40	2.78	9.20
OP 30 (*j* = 3)	5.10	1.30	4.60
OP 40 (*j* = 4)	3.80	0.32	3.90
TOTAL	21.60 (∆*t_μ_*)	3.08 (*σ*)	20.00 (∆*t_theor_*)

**Table 2:** Evaluation of time differences for operations.

	∆*t_j_*		*ε_j_*
OP10 (*j* = 1)	0	<	0.23
OP20 (*j* = 2)	1.20	>	0.92
OP30 (*j* = 3)	0.5	>	0.46
OP40 (*j* = 4)	0.1	<	0.39

**Table 3:** Numerical results.

	Values
Total number of completed cycles (controlled components)	1205
Working cycles requiring more than 20 s	1005
Working cycles requiring less than (or equal to) 20 s	200

**Table 4:** Mean working times in several steps of the framework.

	Value [s]
Theoretical cycle time—∆*t_theor_*	20
Experimental mean cycle time—∆*t_μ_*	21.6
Numerical mean cycle time—∆*t_μ_*_,*s*_	22.4
